# Recombination in the Evolution of Enterovirus C Species Sub-Group that Contains Types CVA-21, CVA-24, EV-C95, EV-C96 and EV-C99

**DOI:** 10.1371/journal.pone.0094579

**Published:** 2014-04-10

**Authors:** Teemu Smura, Soile Blomqvist, Tytti Vuorinen, Olga Ivanova, Elena Samoilovich, Haider Al-Hello, Carita Savolainen-Kopra, Tapani Hovi, Merja Roivainen

**Affiliations:** 1 National Institute for Health and Welfare (THL), Helsinki, Finland; 2 Department of Virology, Haartman Institute, Faculty of Medicine, University of Helsinki, Helsinki, Finland; 3 Department of Virology, University of Turku, Turku, Finland; 4 M.P. Chumakov Institute of Poliomyelitis and Viral Encephalitides, Russian Academy of Medical Sciences, Moscow, Russia; 5 Republican Research and Practical Center for Epidemiology and Microbiology, Minsk, Republic of Belarus; The University of Hong Kong, Hong Kong

## Abstract

Genetic recombination is considered to be a very frequent phenomenon among enteroviruses (Family *Picornaviridae*, Genus *Enterovirus*). However, the recombination patterns may differ between enterovirus species and between types within species. Enterovirus C (EV-C) species contains 21 types. In the capsid coding P1 region, the types of EV-C species cluster further into three sub-groups (designated here as A–C). In this study, the recombination pattern of EV-C species sub-group B that contains types CVA-21, CVA-24, EV-C95, EV-C96 and EV-C99 was determined using partial 5′UTR and VP1 sequences of enterovirus strains isolated during poliovirus surveillance and previously published complete genome sequences. Several inter-typic recombination events were detected. Furthermore, the analyses suggested that inter-typic recombination events have occurred mainly within the distinct sub-groups of EV-C species. Only sporadic recombination events between EV-C species sub-group B and other EV-C sub-groups were detected. In addition, strict recombination barriers were inferred for CVA-21 genotype C and CVA-24 variant strains. These results suggest that the frequency of inter-typic recombinations, even within species, may depend on the phylogenetic position of the given viruses.

## Introduction

Enteroviruses (genus *Enterovirus*, family *Picornaviridae*) are small non-enveloped positive strand RNA viruses with icosahedral capsid symmetry. Enteroviruses are classified to twelve species, *Enterovirus A* to *H*, *J* and *Rhinovirus A* to *C* [1]. Seven of the species, *Enterovirus A* to *D* (formerly named *Human enterovirus A* to *D*) and *Rhinovirus A* to *C* (formerly named *Human rhinovirus A* to *C*) are known to infect humans. Enteroviruses are associated with a wide range of clinical manifestations ranging from mild/sub-clinical respiratory and gastro-intestinal infections to severe central nervous system (CNS) infections [2]. Enteroviruses use faecal-oral and respiratory routes of transmission, and the primary replication site is in the mucosa of the respiratory or gastrointestinal tracts. Occasionally the virus may spread via the lymphatic system and circulation to secondary target tissues such as CNS, heart and pancreas.

Enterovirus genome contains approximately 7500 nucleotides. The genome consists of a single open reading frame (ORF) that is flanked by 5′ end and 3′ end untranslated regions (5′UTR and 3′UTR). The ORF is translated to a single polypeptide that is autocatalytically cleaved to P1, P2 and P3 polyproteins. The P1 polyprotein is further cleaved to capsid proteins VP4 to VP1, whereas P2 and P3 are cleaved to non-structural proteins 2A–2C and 3A–3D, respectively. The non-structural proteins are involved in protein processing, shut-off of host cell translation, host cell membrane rearrangement and viral RNA replication.

On the basis of the capsid coding P1 region, enteroviruses form genetically highly diverse types that are equivalent to serotypes defined by antigenic properties [3], [4]. Enterovirus types are defined by the sequence divergences in the capsid protein VP1 coding region. The members of a same type have more than 75% nucleotide and more than 88% amino acid similarities in the VP1 region [3].

Genetic recombination is a wide spread phenomenon both within (intra-typic recombination) and between (inter-typic recombination) enterovirus types (reviewed in [5]) and recombination events often precede the emergence of novel evolutionary lineages of enteroviruses [6]–[10]. Recombination usually occurs only between members of the same enterovirus species. Evidence for inter-species recombination is limited, although this type of recombination has occurred a few times during enterovirus evolution as judged from the incongruence between phylogenies of 5′UTR and the rest of the genome [11]–[14]. The inter-typic recombination break-points are usually located in the 5′UTR and the non-structural protein coding regions (P2 and P3), whereas recombination seems to be infrequent within the capsid coding P1 region [5], [15], [16]. The frequency of recombination differs between EV-species, being higher in EV-B species than in EV-A species [15] and very low in EV-D species [17], [18]. Within EV-B species there are also type-specific differences in the recombination dynamics [7], [19].

In order to study the inter-typic recombination pattern in EV-C species, we sequenced the partial 5′UTRs of CVA-21, CVA-24, EV-C95, EV-C96 and EV-C99 strains that were isolated during enterovirus surveillance and compared the phylogenies of these strains in 5′UTR and VP1 region. The complete genome of Finnish CVA-24-FIN05-17920 strain was sequenced and analysed along with the complete EV-C genomes retrieved from the GenBank to gain insight into recombination patterns in different parts of the genome.

## Material and Methods

### Ethics statement

The virus samples were collected with the consent of The Institutional Review Board of National Institute for Health and Welfare (THL), the Ethics Committee of M.P. Chumakov Institute of Poliomyelitis and Viral Encephalitides of Russian Academy of Medical Sciences and the Ethical Committee of the Minsk Municipality and analyzed anonymously. The sewage samples were obtained from Viikki wastewater treatment plant in Helsinki, Finland. No specific permission was required for the surveillance for enteroviruses from sewage. The field studies did not involve endangered or protected species.

### Viruses

The virus strains isolated in this study are listed in Table 1. Virus strains were isolated from sewage samples collected during environmental surveillance for polioviruses in Finland using a two-phase concentration method [20]. In addition, clinical enterovirus isolates and isolates from healthy individuals sent to the national enterovirus reference laboratory (National Institute for Health and Welfare, THL) from other Finnish laboratories were included in the study [21]. The rest of the virus strains were received as untypeable nonpolio enteroviruses (NPEV) from a number of National Polio Laboratories of the WHO Polio Laboratory Network supporting the Global Poliovirus Eradication Initiative. Human rhabdomyosarcoma (RD), human colorectal adenocarcinoma (CaCo-2), human cervical carcinoma (HeLa) and green monkey kidney (GMK) cell lines were used for virus isolation.

**Table 1:** The virus strains of which partial 5′UTR was sequenced in this study.

Strain	Collection Date	Country	Sample Type
CVA-21-EST06-E1783-20171_28-Feb-2006	28-Feb-2006	Estonia	Sewage
CVA-21-FIN03-862-36252	2003	Finland	Stool
CVA-21-FIN06-E1906-28163_27-Sep-2006	27-Sep-2006	Finland	Sewage
CVA-21-FIN06-EV06-34A-30796_14-Nov-2006	14-Nov-2006	Finland	Stool
CVA-21-LVA03-756_5-Mar-2003	5-Mar-2003	Latvia	
CVA-21-RUS01-15341_4-Jul-2001	4-Jul-2001	Russia	Stool
CVA-21-SVK05-E1571_Skalica_17-Feb-2005	17-Feb-2005	Slovak Republic	Sewage
CVA-24-AUT05-1600_12-Apr-2005	12-Apr-2005	Austria	
CVA-24-FIN02-671-2002	2002	Finland	Sewage
CVA-24-FIN03-817-1619-2003	2003	Finland	Stool
			
CVA-24-FIN04-6B-2004	2004	Finland	Stool
CVA-24-FIN04-EV04-27A-2124	2004	Finland (India)	Stool
CVA-24-FIN04-EV04-34A-3787	2004	Finland (India)	Stool
CVA-24-FIN05-1-7920-_4-Jan-2005	4-Jan-2005	Finland (China)	Stool
CVA-24-FIN05-1663-13794_18-Oct-2005	18-Oct-2005	Finland	Stool
CVA-24-FIN05-EV05-17A_31-Oct-2005	31-Oct-2005	Finland (China)	Stool
CVA-24-FIN06-1869-25202_6-Sep-2006	6-Sep-2006	Finland	Stool
			
CVA-24-FIN06-EV06-32A-30807_24-Oct-2006	24-Oct-2006	Finland	Stool
CVA-24-FIN06-EV07-2A-29392_18-Dec-2006	18-Dec-2006	Finland	Stool
CVA-24-LVA03-757_19-Jun-2003	19-Jun-2003	Latvia	
			
CVA-24-RUS-00-14038_21-Nov-2000	21-Nov-2000	Russia	Stool
CVA-24-RUS-01-14455_14-Feb-2001	14-Feb-2001	Russia	Stool
CVA-24-RUS-TKM01-14868_10-Mar-2001	10-Mar-2001	Russia (Turkmenistan)	Stool
CVA-24-RUS-KGZ01-15071_18-Jun-2001	18-Jun-2001	Russia (Kyrgyzstan)	Stool
CVA-24-RUS-UZB01-15213_20-Jun-2001	20-Jun-2001	Russia (Uzbekistan)	Stool
EV-C96-FIN05-5-2005	2005	Finland (China)	Stool
EV-C96-FIN05-12-2005	2005	Finland (China)	Stool
EV-C96-FIN06-7-2006	2006	Finland (China)	Stool
EV-C96-FIN06-9-2006	2006	Finland	Stool
EV-C96-SVK03-24-2003	2003	Slovak Republic	Stool
EV-C99-BLR00-32864-2000	2000	Republic of Belarus	Stool
EV-C99-BLR00-32878-2000	2000	Republic of Belarus	Stool
EV-C99-BLR00-32881-2000	2000	Republic of Belarus	Stool
EV-C99-BLR00-32887-2000	2000	Republic of Belarus	Stool
EV-C99-BLR00-33291-2000	2000	Republic of Belarus	Stool
EV-C99-BLR00-33405-2000	2000	Republic of Belarus	Stool
EV-C99-BLR00-33483-2000	2000	Republic of Belarus	Stool
EV-C99-FIN06-EV06-31B-30779_6-Nov-2006	6-Nov-2006	Finland	Stool
EV-C99-RUS-TKM00-13831_8-Sep-2000	8-Sep-2000	Russia (Turkmenistan)	Stool
EV-C99-SVK03-23-20226_7-Feb-2003	7-Feb-2003	Slovak Republic	
EV-C99-SVK04-E1152-44722_19-May-2004	19-May-2004	Slovak Republic	Sewage

### Partial VP1 and 5′UTR RT-PCR and sequencing

Viral RNA was extracted from infected cell cultures with RNeasy Total RNA kit (Qiagen, Hilden, Germany) or E.Z.N.A. Total RNA Kit Omega (Bio-Tek Inc., Doraville, GA, USA) according to the manufacturer's instructions. RT-PCR was carried out as described previously [22] using primers 292 and 222. For 5′UTR RT-PCR, primers (FWD-TTAAAACAGCCTGTGGGTTG and REV- CCCAAAGTAGTCGGTTCCGC) were used. PCR amplicons were purified with the QIAquick gel extraction kit (Qiagen). Sequencing reactions with BigDye Terminator cycle sequencing ready reaction kit v3.1 (Life Technologies, Carlsbad, CA, USA) and sequencing with ABI3730 Automatic DNA Sequencer (Life Technologies) were performed by Institute for Molecular Medicine Finland (FIMM) Sequencing Laboratory. The electropherograms were analysed using Geneious Pro 6.0 software (Biomatters Ltd, Auckland, New Zealand, http://www.geneious.com).

### Full-length genome sequencing of CVA-24-FIN05-1-7920 strain

Since the CVA-24-FIN05-1-7920 strain did not form clearly distinguishable plaques in Hela cells, it was purified using the end-point titration method in the Ohio strain of HeLa cells. Subsequently, the virus was passaged once in HeLa cells at 36°C, freeze–thawed three times and clarified by centrifugation at 250 g for 10 min.

Total RNA was extracted from infected cells using Trizol reagent (Gibco-BRL Life Technologies) according to the manufacturer's instructions. SuperScript First-strand Synthesis System for RT-PCR kit (Gibco-BRL Life Technologies) was used for cDNA synthesis and SuperScript One-step RT-PCR for Long Templates kit was used for PCRs. PCR products were purified using QIAEXII agarose gel extraction kit (Qiagen). The complete genome of the virus was sequenced using primer-walking strategy.

### Sequence dataset collection

For the recombination analysis, the complete genome sequences of the EV-C species were obtained from GenBank with search terms ‘complete genome’, ‘enterovirus’, ‘coxsackievirus’ and ‘poliovirus’ (search 7.2.2013) and analysed with the complete genome sequence of strain CVA-24-FIN05-1-7920. To relieve computational demands and correct data collection bias, only one representative sequence from the clusters that shared more than 95% similarity with each other was included in the analysis.

The second dataset contained partial 5′UTRs sequenced in this study, the overlapping sequences of Enterovirus C prototype strains and all sequences with more than 95% similarity in BLAST search (14.12.2012) with the 5′UTR of EV-C96, CVA-21, EV-C99 or CVA-24 strains as query sequences. The phylogeny of 5′UTR dataset was compared to the phylogeny of partial VP1 sequences described in detail elsewhere [23].

### Sequence analysis

The sequences were aligned using ClustalW algorithm implemented in MEGA version 5.05 [24] followed by manual refinement. Phylogenetic trees were constructed using neighbor-joining (NJ) method implemented in MEGA version 5.05. Bootstrap resampling with 1000 replicates was conducted. Various substitution models including Tamura-Nei (TN93) [25] and general time reversible (GTR) [26] models were used.

The rates of evolution and divergence times of the virus lineages were estimated using Bayesian MCMC method implemented in BEAST version 1.7.4 [27]. The analyses were performed using a relaxed molecular clock model (the uncorrelated log-normal distributed model) [28], GTR model of substitution and Bayesian skyline demographic model. The Bayesian analyses were run for 100 million states and sampled every 1000 states. The analyses were carried out on the Bioportal server, University of Oslo (www.bioportal.uio.no) [29] and in CSC – IT Center for Science Ltd. (Espoo, Finland). The analyses were run in duplicate and the log-files were combined to increase the effective sample size. Posterior probabilities were calculated with a burn-in of 10 million states and checked for convergence using Tracer version 1.5. [30].

The SimPlot 3.5.1 program was used for similarity plot and bootscanning analysis [31]. For similarity plot analysis, a 200-nt window moved in 20-nt steps was used. For the bootscanning analysis [32] a 500-nt window moved in 20-nt steps and NJ-algorithm run with 100 pseudoreplicates were used.

TreeOrder scan [15] implemented in SSE 1.6 program [33] was used for EV-C complete genome segregation analysis. Fragments of 500 nt moved in 100 nt steps and NJ-algorithm run with 100 bootstrap psudoreplicates was used for this analysis. A bootstrap value of 70 was used as the threshold for scoring phylogeny violations.

### GenBank accession numbers

The GenBank accession numbers for the sequenced strains are KJ152640- KJ152683 and KF128985–KF129056.

## Results

### Phylogenetic incongruity between 5′UTR and VP1 of EV-C sub-group B

**Figure 1:**
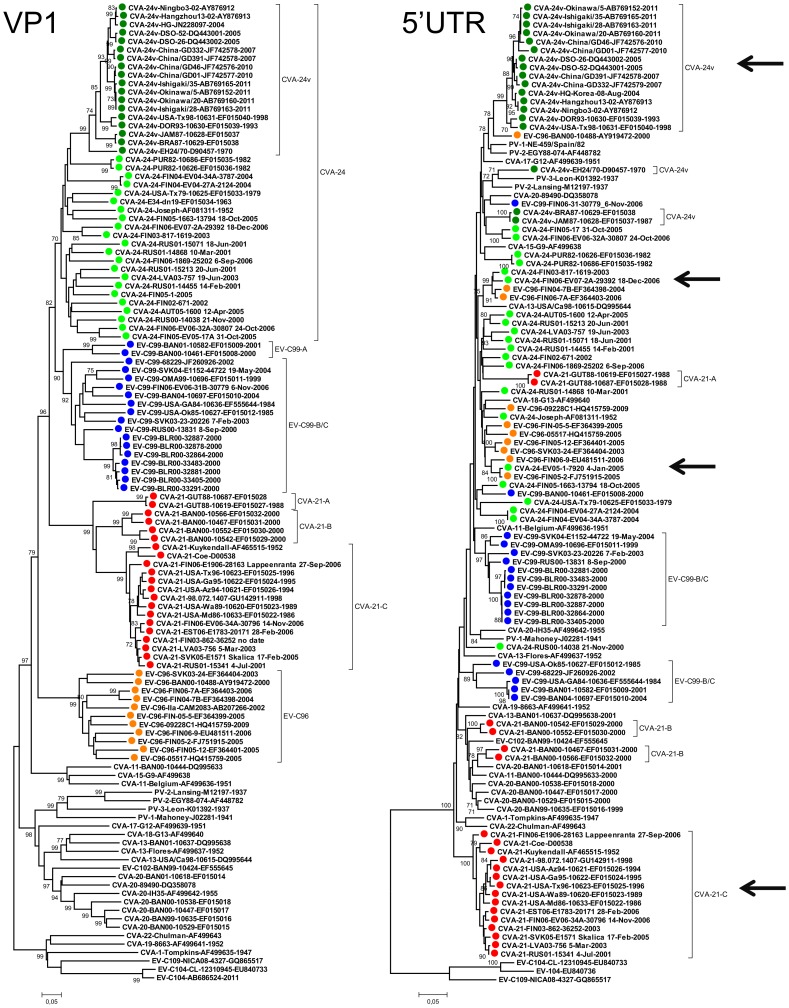
The VP1 and 5′UTR phylogeny of EV-C species. A phylogenetic tree constructed from partial VP1 (343 nucleotides) (left) and 5′UTRs (the first 540 nucleotides of consensus alignment) (right) of CVA-21, CVA-24, EV-C95, EV-C96 and EV-C99 strains, EV-C prototype strains and strains retrieved from the GenBank that had more than 95% similarity in 5′UTR with the strains sequenced in this study. The types of the strains (defined as more than 75% nucleotide and more than 88% amino acid similarities in the VP1 region [3], [4]) are indicated with colours (CVA-21 red; CVA-24 green; CVA-24v dark green; EV-C96 orange; EV-C99 blue). Sub-trees that are shown in Fig 2 are indicated with arrows. The trees were constructed using the Neighbour-Joining method and the Tamura-Nei substitution model. The bootstrap support values were calculated for 1000 replicates. The bootstrap support values >70 are shown.

In the VP1 coding region, EV-C types cluster into three sub-groups [23], [34]–[37] which were designated here as A to C. To gain insight into the recombination history of EV-C subgroup B, partial 5′UTR regions (corresponding to the first 540 nucleotides of the consensus alignment) of a set of EV-C96, CVA-21, EV-C99 and CVA-24 strains (Table 1) were sequenced and analysed together with the sequences retrieved from the GenBank. The phylogeny of 5′UTR was compared to that of VP1 sequences (Fig 1).

**Figure 2:**
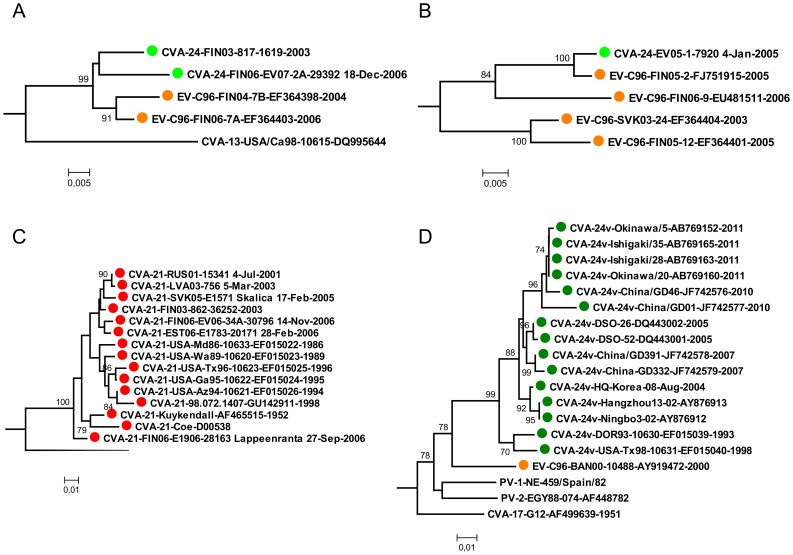
Sub-trees that are indicated with arrows in Figure 1. (a–b) Sub-trees of strains that showed inter-typic recombination events. (c) Sub-tree constructed from CVA-21 genotype C strains. (d) Sub-tree constructed from CVA-24v strains. The trees were constructed using the Neighbour-Joining method and the Tamura-Nei substitution model. The bootstrap support values were calculated for 1000 replicates. The bootstrap support values >70 are shown.

In the 5′UTR, the EV-C96, CVA-21, EV-C99 and CVA-24 strains formed several sub-clusters that were not equivalent to corresponding types (i.e. VP1 clusters) (Fig 1). This incongruent clustering pattern between 5′UTR and VP1 regions suggested several inter-typic recombination events between the virus strains. Clear indication of such recombination events were detected between the ancestor of CVA-24-FIN06-EV-07-2A-29392 and CVA-24-FIN03-817-1619 and the ancestor of EV-C96-FIN06-7A and EV-C96-FIN04-7B (Fig 2a) and between CVA-24-FIN05-1-7920 and EV-C96-FIN05-2 (Fig 2b).

There were two notable exceptions to the apparently high inter-typic recombination frequency. The strains of CVA-21 genotype C formed a monophyletic group in both 5′UTR and VP1 coding region (Fig 1, Fig 2c). Notably, this genotype contained the prototype strain CVA-21-Kuykendal and the strain CVA-21-Coe (both isolated in the 1950′s), suggesting that no inter-typic recombination events has occurred in this genotype for at least six decades. In contrast, the strains of CVA-21 genotype B that contained four strains isolated in Bangladesh clustered together with several other EV-C strains isolated during the same time period in Bangladesh [35]. The two sequences of CVA-21 genotype A did not group together with any of the currently known 5′UTR sequences.

Likewise, most of the CVA-24 variant (CVA-24v) strains that cause acute haemorrhagic conjunctivitis formed monophyletic groups in both VP1 and 5′UTR (Fig 2d). However, in 5′UTR this cluster excluded the variant strains CVA-24-BRA87 and CVA-24-JAM87 isolated in Brazil and Jamaica in 1987 [35] and the first known AHC-causing CVA-24 variant strain EH24/70 isolated in 1970 [38]. CVA-24-EH24/70 showed some resemblance (i.e. formed a group with ∼70 bootstrap value) with PV prototype strains PV-2-Lansing and PV-3-Leon, whereas the majority of CVA-24v strains showed resemblance with EV-96-BAN00-10488, PV-2-EGY88-074 and PV-1-NE-459/Spain/82-L76409 (Fig 2d).

To estimate the time of these recombination events, the evolutionary rates and dates of most recent common ancestors (tMRCA) for these 5′UTR clusters were estimated using Bayesian MCMC method with relaxed molecular clock (Table 2). Essentially similar tMRCAs were estimated using GTR and TN substitution models including or excluding gamma rated variability between sites. The estimated time of recombination event for CVA-24-FIN05-1-7920 and EV-C96-FIN05-2 was about three years prior to isolation of these virus strains. The estimated MRCA for CVA-21-C clade dated about 70 years ago whereas the MCRA of the currently circulating CVA-24v lineage emerged most likely in the early 1980′s.

**Table 2:** The estimated tMRCAs (year) for 5′UTR recombinant clusters in EV-C sub-group B.

	GTR		GTR+G		TN		TN+G	
	tMRCA	95% HPD	tMRCA	95% HPD	tMRCA	95% HPD	tMRCA	95% HPD
CVA-24-FIN05-1/EV-C96-FIN05-2	2002	1999-2005	2002	1999–2005	2002	1998–2005	2002	1999–2005
EV-C96-FIN06-7/FIN04-7/CVA-24-FIN06-EV07-2A-29392/FIN03-817-1619	1992	1983–2000	1994	1986–2001	1991	1981–2000	1994	1985–2001
CVA-21-C	1939	1928–1948	1940	1930–1949	1937	1924–1948	1940	1930–1949
CVA-24v	1983	1985–1991	1985	1976–1992	1981	1969–1990	1985	1976–1992
Mean Rate (x10^−3^)	1.139	0.858–1.435	1.996	1.401–2.619	1.059	0.790–1.345	2.028	1.413–2.671
Coefficient of variation	0.698	0.532–0.875	0.758	0.553–0.977	0.680	0.518–0.853	0.735	0.535–0.947

The analysis was conducted using BEAST program. Tamura-Nei and GTR models of substitution, with or without gamma rated variability between sites (G), lognormal relaxed clock and Bayesian Skyline demographic model were used in the analysis.

### Complete genome analysis of the CVA-24-FIN05-1-7920 strain

Since the strains CVA-24-FIN05-1-7920 and EV-C96-FIN05-2 had >99% nt similarity in 5′UTR and an apparently recent recombination origin, the complete genome of CVA-24-FIN05-1-7920 was sequenced and analysed along with EV-C96-FIN05-2 strain sequenced previously [39] and the complete genome sequences of EV-C strains retrieved from the GenBank. Both strains CVA-24-FIN05-1-7920 and EV-C96-FIN05-2 were isolated in Turku, Finland, in January 2005 from stool samples of healthy children adopted from China.

**Figure 3:**
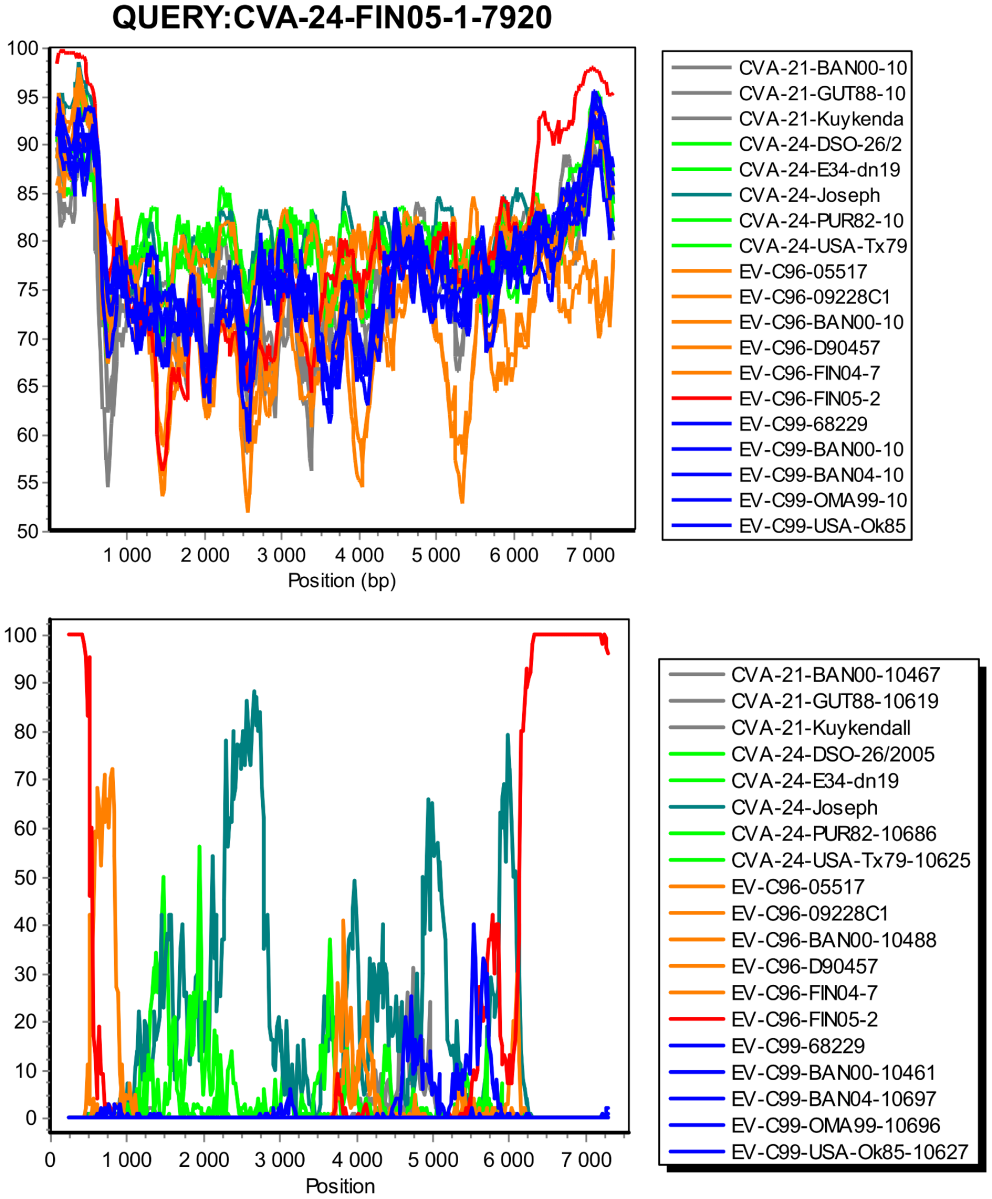
Complete genome similarity plot and bootscanning analyses. The similarity plot (a) and bootscanning (b) analysis of those CVA-21, CVA-24, EV-C96 and EV-C99 strains, of which a complete genome sequence was available (GenBank search 7.2.2013). The similarity plot analysis was conducted using a sliding window of 200 nt moving in 20 nt steps with CVA-24-FIN05-1-7920 as a query sequence. The bootscanning analysis was conducted using a sliding window of 500 nt moving in 20 nt steps. The genetic distances were computed with the Kimura 2-parameter nucleotide substitution model, and Neighbour-Joining method was used for the phylogenetic tree construction. The bootstrap values were calculated for 100 replicates. The transition to transversion ratio (Ts/Tv) was estimated for each window.

The similarity plot and bootscanning analyses suggested that CVA-24-FIN05-1-7920 shared high sequence similarity and high bootstrap support for clustering with EV-C96-FIN05-2 in the 5′UTR and the 3′ end of the genome (approx. 1000 nt, consisting the most of the 3D polymerase gene) (Fig. 3).

As expected, in the capsid coding P1 region CVA-24-FIN05-1-7920 showed similarity to other CVA-24 strains. In the highly conserved VP4 coding region the phylogeny was unresolved (Fig S1a), whereas in the capsid coding VP2, VP3 and VP1 regions the strain CVA-24-FIN05-1-7920 grouped together with other CVA-24 strains (Fig S1b-d). However, none of the currently known CVA-24 strains had >82% similarity with this strain in VP2-VP1 coding regions (BLAST search 22.2.2013). Likewise, although the strain CVA-24-FIN05-1-7920 grouped together with the other strains of EV-C sub-group B in the P2 region (Fig S1e-i), no close relatives (i.e. >81% sequence similarity) were found for this strain in the 2A–3C protein coding regions. In 3D coding region, CVA-24-FIN05-1-7920 clustered together with EV-C96-FIN05-2 (Fig S1j).

### Recombination analysis of EV-C species complete genomes

**Figure 4:**
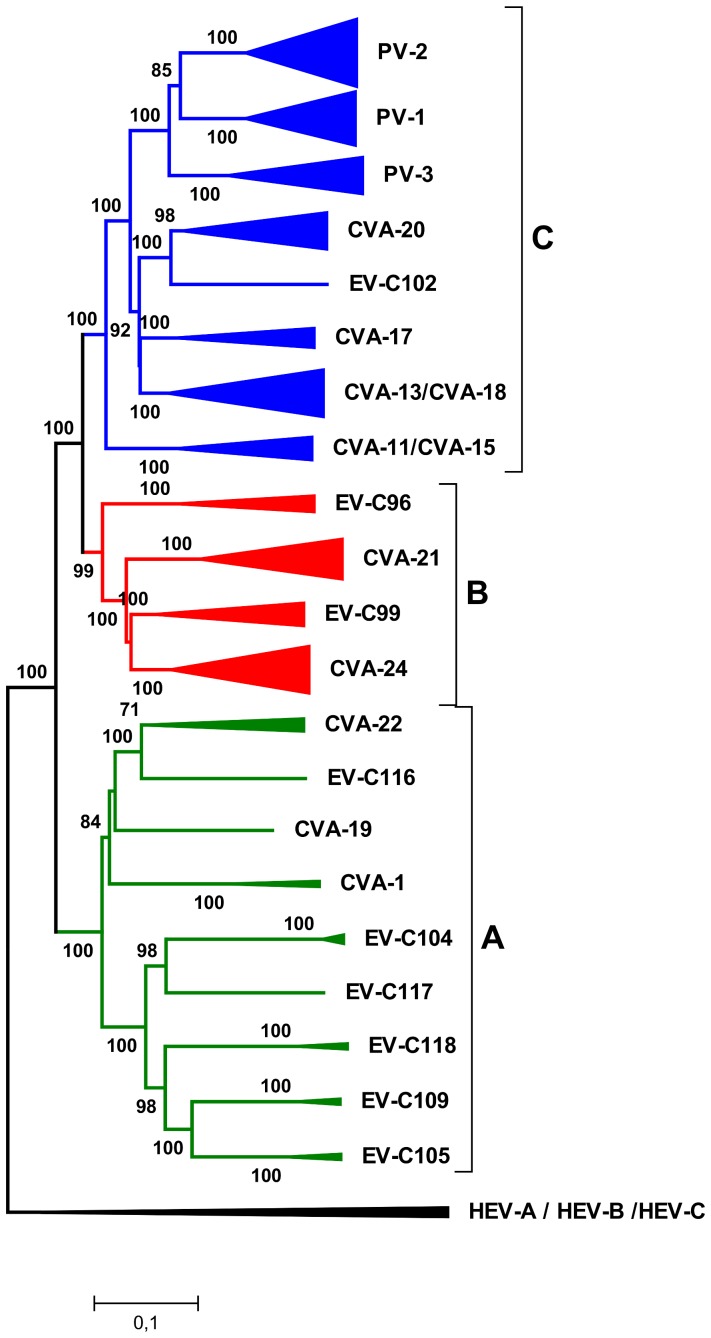
The phylogeny of EV-C P1 region. The phylogenetic trees constructed from the P1 coding regions of EV-C strains, of which a complete genome sequence was available (GenBank search 27.2.2013). The trees were constructed using the Neighbour-Joining method and the Tamura-Nei substitution model. The bootstrap support values were calculated for 1000 replicates. The bootstrap support values >70 are shown. The strains of EV-C sub-groups A, B and C are shown in green, red and blue, respectively.

**Figure 5:**
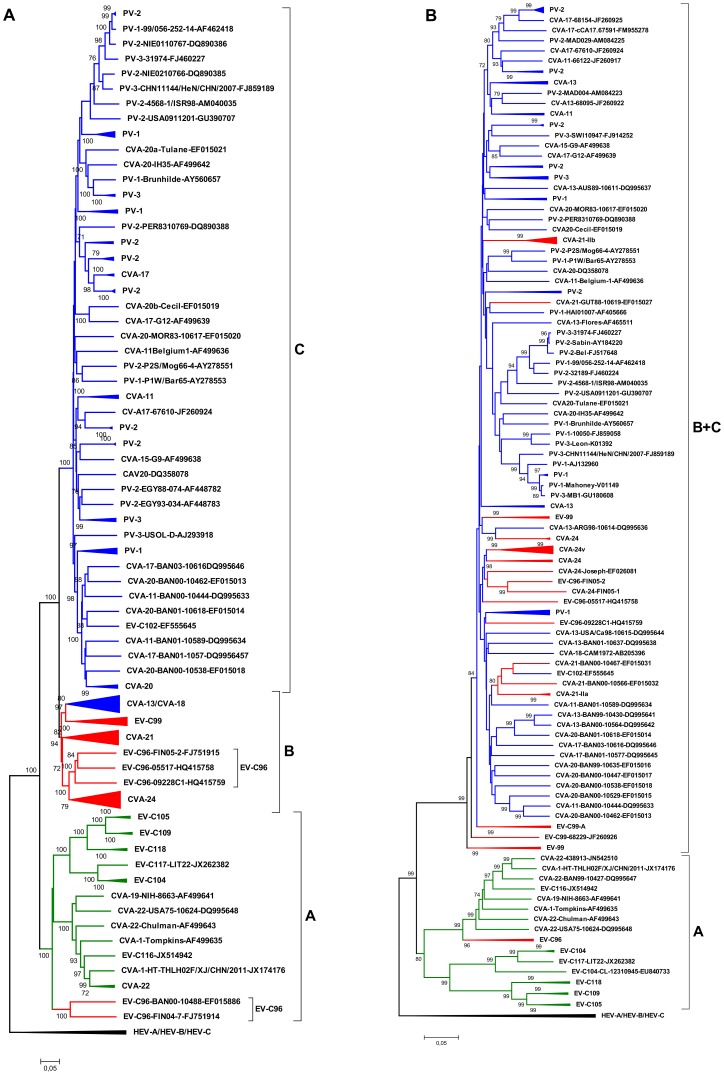
The phylogeny of EV-C P2 and P3 regions. The phylogenetic trees constructed from the (a) P2 and (b) P3 coding regions of EV-C strains, of which a complete genome sequence was available (GenBank search 27.2.2013). The trees were constructed using the Neighbour-Joining method and the Tamura-Nei substitution model. The bootstrap support values were calculated for 1000 replicates. The bootstrap support values >70 are shown. The strains of EV-C sub-groups A, B and C are shown in green, red and blue, respectively.

Notably, the three major P1 sub-groups of EV-C species (A–C; designated on the basis of P1 phylogeny, see above) remained congruent in the P2 region (Fig 4, Fig 5a). There were only two exceptions: the CVA-13/18 strains of the P1 sub-group A grouped together with EV-C99 in the EV-C P2 sub-group B and, as described previously [35], [39], EV-C96 strains FIN04-7 and BAN00-10488 of the P1 sub-group B grouped together with the strains of the EV-C P2 sub-group A. However, within each of the EV-C sub-groups (A–C), the P2 phylogenies were largely incongruent with those of the P1 region, suggesting promiscuous recombination within EV-C sub-groups A, B and C (in contrast to the apparently rare recombination between these sub-groups). An exception to this was the EV-C sub-group A1 that contained EV-C104, EV-C105, EV-C107, EV-C109 and EV-C118 that showed no evidence of inter-typic recombination between the P1 and P2 regions.

In the P3 region, all of the strains from EV-C sub-groups B and C merged into one large cluster (B+C), whereas EV-C sub-group A remained separate from the others (Fig. 5b). Only a few P1 clusters remained congruent throughout the genome. Two notable exceptions were presented by the strains of CVA-21 genotype C and CVA-24v (AHC-causing variant) strains, which (in accordance with results obtained from 5′UTR) showed no traces of inter-typic recombination.

**Figure 6:**
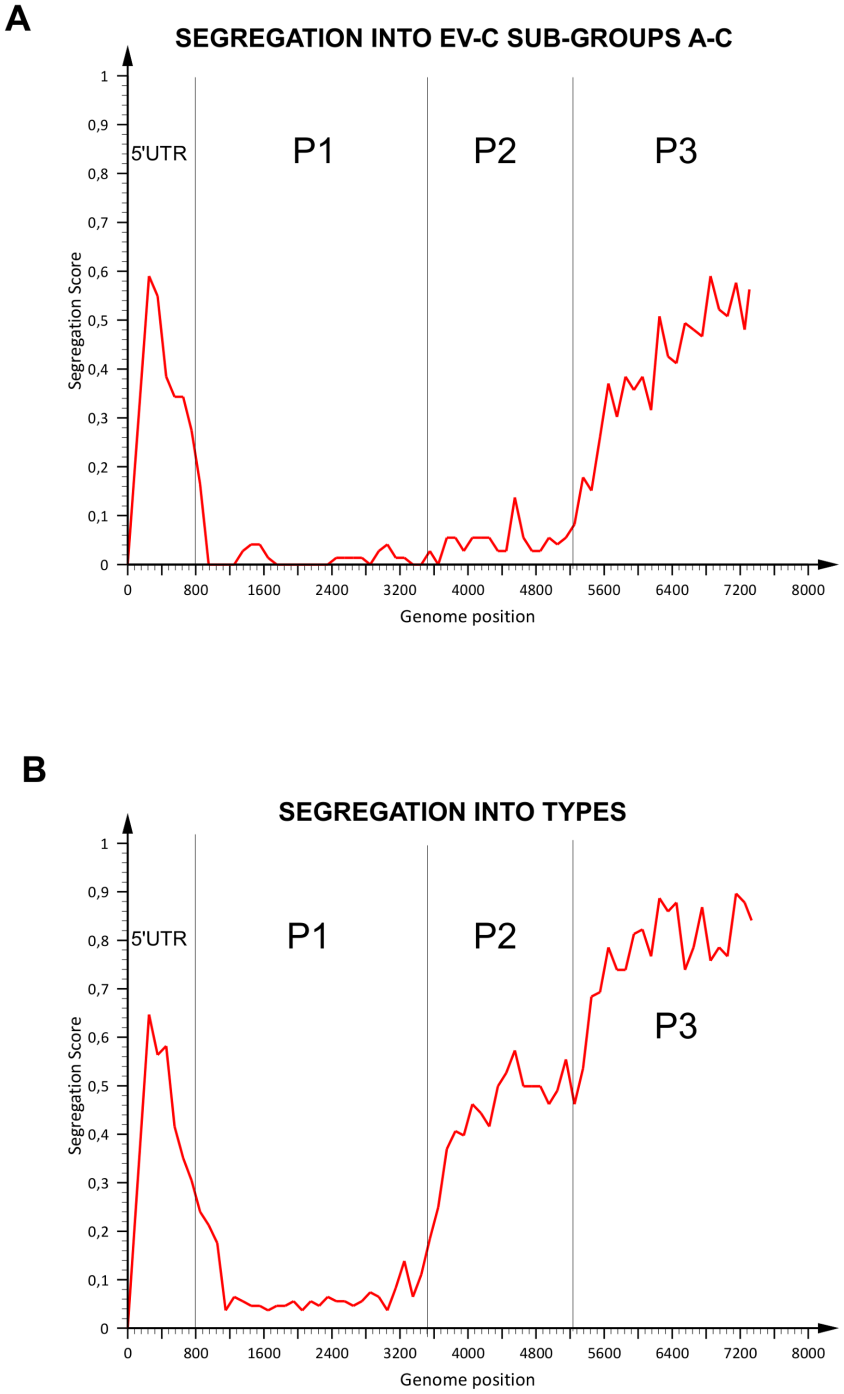
Segregation of EV-C strains into pre-defined groups. Correspondence between the phylogeny of sequential fragments of 500 nucleotides throughout the enterovirus genome and the assignment of EV-C strains into sub-groups A, B and C (a) and types (b). Correspondence between phylogeny and EV-C sub-group/type is indicated by the concordance of the sub-group/type assignment with phylogenetic ordering; segregation score of 0 corresponds to trees where strains are invariably segregated into pre-defined groups (i.e EV-C sub-group A, B and C (a) or types (b)), while 1 corresponds to phylogenetic trees where strains were randomly distributed throughout the tree.

The segregation of EV-C strains into the three major EV-C sub-groups (A to C) in different genomic regions was further evaluated using Tree order scan method [15]. This analysis measures the correspondence between the phylogeny of the sequences in the given genome fragment and their pre-assigned groups. The EV-C strains were labelled according to their P1 sub-groups (A–C), and phylogenetic trees were constructed from sequentially generated genome fragments of 500 nucleotides moved in 100 nt steps. Segregation values were plotted as values from 0 (strains perfectly segregated into EV-C sub-groups A–C) to 1 (strains randomly distributed) (Fig. 6).

In the P1–P2 region, from approximate nucleotide positions 900 to 5200 in the alignment, sequences segregated according to EV-C sub-groups A–C, with segregation scores at or close to 0 (Fig 6a). In the 5′UTR-VP4 junction (corresponding to nucleotides 700–900 in the consensus alignment), there was sharp transition to higher segregation value representing more random tree orders. Similar transition point towards higher segregation value was located between nt 5200–5800 corresponding to 3A–3C proteins in P3. In general, these transition points corresponded to the phylogenetic trees constructed on the basis of P1, P2 and P3 regions.

When segregation into types (instead of sub-groups) was studied, the low segregation values were more strictly restricted to P1 region (nucleotides 779–3464), whereas step-wise increase in the segregation values were observed in P2 (nucleotide 3464–5192) and P3 (nucleotides 5200–7534) (Fig 6b).

## Discussion

Enteroviruses, in general, have high rates of recombination both within and between types [5], [15], [40]. However, the frequency of recombination differs between EV-species, being higher in EV-B species than in EV-A species [15] and very low in EV-D species [17], [18]. Within EV-B species also type-specific differences in the recombination dynamics have been documented [7]. In the present study, type- and genotype-specific differences in the recombination patterns between EV-C strains were detected.

The results of the complete genome analysis of EV-C strains suggested that, in the P2 region, recombination has occurred mainly between the strains that are closely related in the P1 region (i.e. within EV-C sub-groups A, B and C). In the P2 region, the strains formed a clustering pattern where deep nodes of the tree were generally congruent to those of the P1 tree, but more recent type-specific nodes were not. This suggests that in this region recombination is rare between the EV-C sub-groups A, B and C, but rather frequent within these groups. However, the two exceptions for this clustering pattern, CVA-13 and the two EV-C96 strains, suggest that the recombination barrier between the EV-C sub-groups can be crossed (i.e., there is no strict incompatibility between the P1 and P2 regions of EV-C sub-groups A, B and C). Apparently, crossing the recombination barrier occurs infrequently because CVA-13 strains and the two EV-C96 strains (BAN00-10488 and FIN04-7) remained as separate clusters within EV-C sub-groups A and B at the P2 region, suggesting that the respective recombination events are most likely ancient. EV-C sub-groups B and C merged together in the P3 region, suggesting frequent inter-typic recombination between the types of these sub-groups at the 3′ end of the genome. In contrast, the strains of EV-C sub-group A showed only restricted recombination with the strains of EV-C sub-groups B and C in all regions of the genome, as suggested previously [37].

In the 5′UTR the strains did not cluster according to the P1 grouping pattern, but formed two major clusters. In accordance with the previous studies [13], [37], [41]–[43], the types EV-C104, EV-C105, EV-C109, EV-C117 and EV-C118 formed a cluster distinct from other EV-C types in this region. Several recombination events were inferred by the phylogenetic incongruity between 5′UTR and VP1 coding region. Notably, all of the recombination events detected in the strains sequenced in this study had occurred between the strains of EV-C sub-group B (i.e between EV-C96 and CVA-24). This suggests that there may be a preference for recombination between these closely related virus types. Alternatively, such recombination pattern may arise due to biased sequence dataset. More studies are needed to elucidate the potential recombination preferences of different EV-C types.

In addition to loosely restricted recombination between the EV-C sub-groups, a strict recombination barrier was observed between the CVA-24v and other EV-C strains both in complete genome and 5′UTR analyses. In agreement with previous studies [35], [44], CVA-24v strains clustered together in all coding regions of the genome suggesting that no recombination between the CVA-24v strains and other EV-C strains has occurred in the coding region of the genome since the emergence of the CVA-24v lineage in the year 1970 [45]. Likewise, most of the CVA-24v strains formed a monophyletic group in 5′UTR. However, some of the earliest CVA-24v strains (including the first isolate CVA-24-EH24/70) did not cluster together with the modern CVA-24v strains. Therefore, a possibility of infrequent recombination in 5′UTR of CVA-24v strains cannot be excluded.

A strict recombination barrier was also detected for the strains of CVA-21 genotype C. The strains of this genotype group together at P1, P2, P3 (Fig. 4; Fig 5) and the 5′UTR (Fig 1). The recombination barrier seems to be specific to genotype C of CVA-21, because the strains of CVA-21 genotypes A and B have apparently recombined with other EV-C viruses in the 5′UTR and P3 regions (Fig. 1; Fig 5). Notably, in addition to recently isolated strains, the CVA-21 genotype C contained the strains Kuykendall and Coe isolated in the 1950′s and the MCMC-analysis suggested that the common ancestor of these strains has emerged between the late 1930′s and early 1940′s, suggesting that recombination has not occurred in this genotype during the past 60–70 years.

Such strict recombination barriers are unusual for enteroviruses that in general are known for promiscuous recombination [15]. The co-infection of a certain cell is a pre-requisite for recombination, and different tissue tropism might efficiently restrict recombination. Therefore, the tissue tropism of CVA-24v and CVA-21 (i.e. conjunctiva and respiratory tract, respectively) might restrict their recombination with other EV-C types, which are primarily intestinal pathogens. Notably, the strains of CVA-21 genotypes A and B have been isolated from stool samples [35], [46] suggesting that there may be intra-typic differences in the tissue tropism of CVA-21 genotypes. Likewise, tissue tropism may partly account for the recombination barrier between the types of EV-C sub-group A and B/C, since many, but not all, of the sub-group A serotypes have been isolated from the respiratory tract [13], [41]–[43], [47]–[49].

Other factors that may affect recombination frequency include viral prevalence, geographical differences in the occurrence of viruses, sufficient nucleotide similarity, co-operation of cis-acting elements within the genome, the functionality of the resulting chimeric proteins and the co-operation of mature proteins. Because homologous recombination requires substantial sequence similarity between recombination partners [50], recombination is expected to be more common between close relatives and become gradually less common with more divergent strains. Furthermore, if nucleotide similarity increases recombination frequency, a positive feedback effect, where a recombination event increases the probability of further recombinations, would be expected. This could be an explanation for the loosely restricted recombination in the P2 region of EV-C sub-group B, and lead to a phenomenon where the inter-typic recombination frequency is at least partially dependent on the phylogenetic positions of the given viruses.

Altogether, the phylogenetic pattern of the EV-C strains suggests non-random recombination between strains of the same species. Therefore, the frequency of recombination appears to differ between species [15], types and intra-species groupings, which is possibly due to cell tropism, sequence similarity and the phylogenetic position of a given virus strain.

## Supporting Information

Figure S1 **The phylogenetic trees constructed from distinct genes of CVA-24-FIN05-1-7920 and other EV-C strains, of which a complete genome sequence was available.** The nodes with CVA-24-FIN05-1-7920 are shown as sub-trees. The trees were constructed using the Neighbour-Joining method and the Tamura-Nei substitution model. The bootstrap support values were calculated for 1000 replicates. The bootstrap support values >70 are shown.
(PDF)
