# Supplementary material for: Recombination in the Evolution of Enterovirus C Species Sub-Group that Contains Types CVA-21, CVA-24, EV-C95, EV-C96 and EV-C99
Source: PLoS One. 2014 Apr 10;9(4):e94579. doi: 10.1371/journal.pone.0094579 (PMC3983234; doi:10.1371/journal.pone.0094579)
Supplement: Figure S1 — The phylogenetic trees constructed from distinct genes of CVA-24-FIN05-1-7920 and other EV-C strains, of which a complete genome sequence was available. The nodes with CVA-24-FIN05-1-7920 are shown as sub-trees. The trees were constructed using the Neighbour-Joining method and the Tamura-Nei substitution model. The bootstrap support values were calculated for 1000 replicates. The bootstrap support values >70 are shown. (PDF) [file pone.0094579.s001.pdf]

(a) VP4

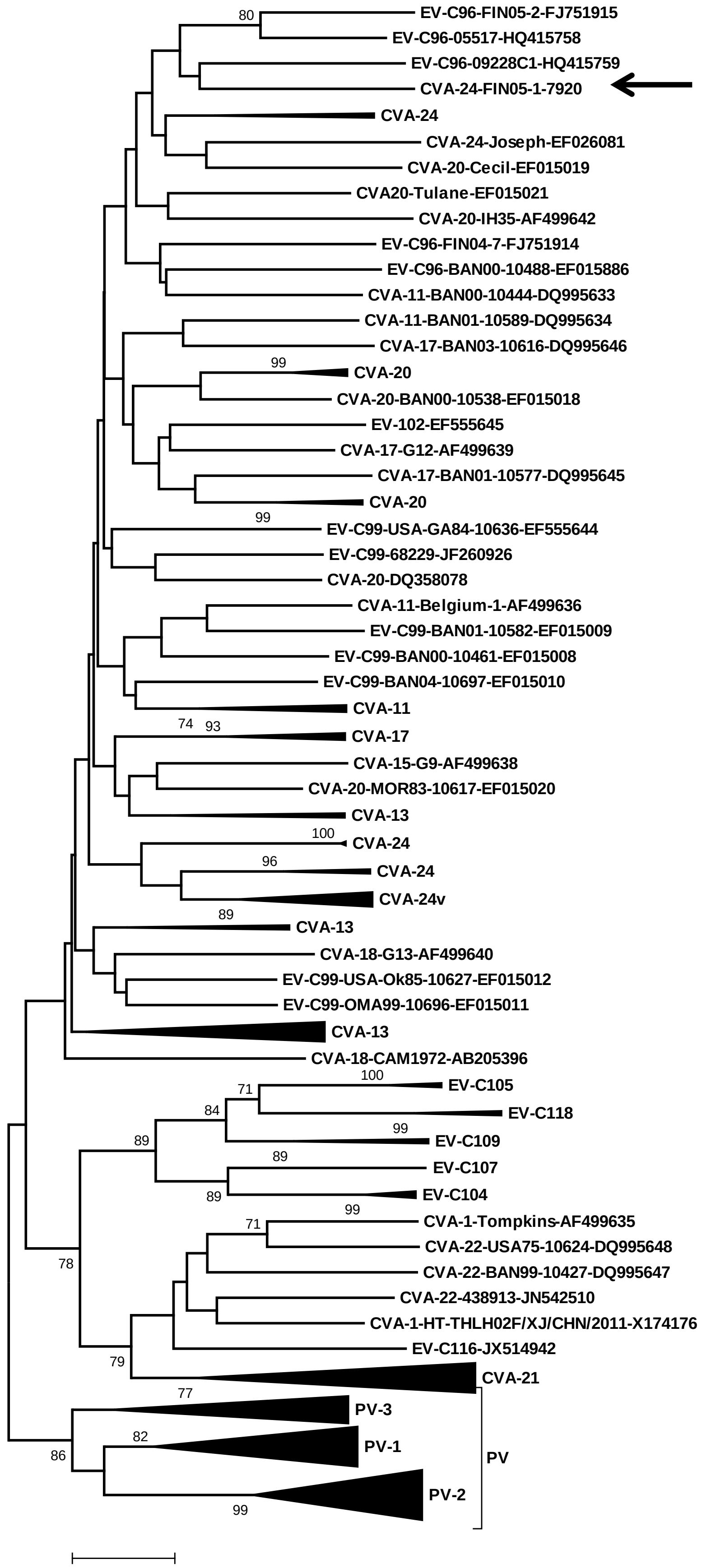

80
EV-C96-FIN05-2-FJ751915
EV-C96-05517-HQ415758
EV-C96-09228C1-HQ415759
CVA-24-FIN05-1-7920
CVA-24
CVA-24-Joseph-EF026081
CVA-20-Cecil-EF015019
CVA20-Tulane-EF015021
CVA-20-IH35-AF499642
EV-C96-FIN04-7-FJ751914
EV-C96-BAN00-10488-EF015886
CVA-11-BAN00-10444-DQ995633
CVA-11-BAN01-10589-DQ995634
CVA-17-BAN03-10616-DQ995646
99
CVA-20
CVA-20-BAN00-10538-EF015018
EV-102-EF555645
CVA-17-G12-AF499639
CVA-17-BAN01-10577-DQ995645
CVA-20
99
EV-C99-USA-GA84-10636-EF555644
EV-C99-68229-JF260926
CVA-20-DQ358078
CVA-11-Belgium-1-AF499636
EV-C99-BAN01-10582-EF015009
EV-C99-BAN00-10461-EF015008
EV-C99-BAN04-10697-EF015010
CVA-11
74
93
CVA-17
CVA-15-G9-AF499638
CVA-20-MOR83-10617-EF015020
CVA-13
100
CVA-24
96
CVA-24
CVA-24v
89
CVA-13
CVA-18-G13-AF499640
EV-C99-USA-Ok85-10627-EF015012
EV-C99-OMA99-10696-EF015011
CVA-13
CVA-18-CAM1972-AB205396
100
71
EV-C105
84
EV-C118
89
99
EV-C109
89
EV-C107
89
EV-C104
99
71
CVA-1-Tompkins-AF499635
CVA-22-USA75-10624-DQ995648
CVA-22-BAN99-10427-DQ995647
78
CVA-22-438913-JN542510
CVA-1-HT-THLH02F/XJ/CHN/2011-X174176
EV-C116-JX514942
79
CVA-21
77
PV-3
82
PV-1
86
PV
PV-2
99
0,05

(b) VP2

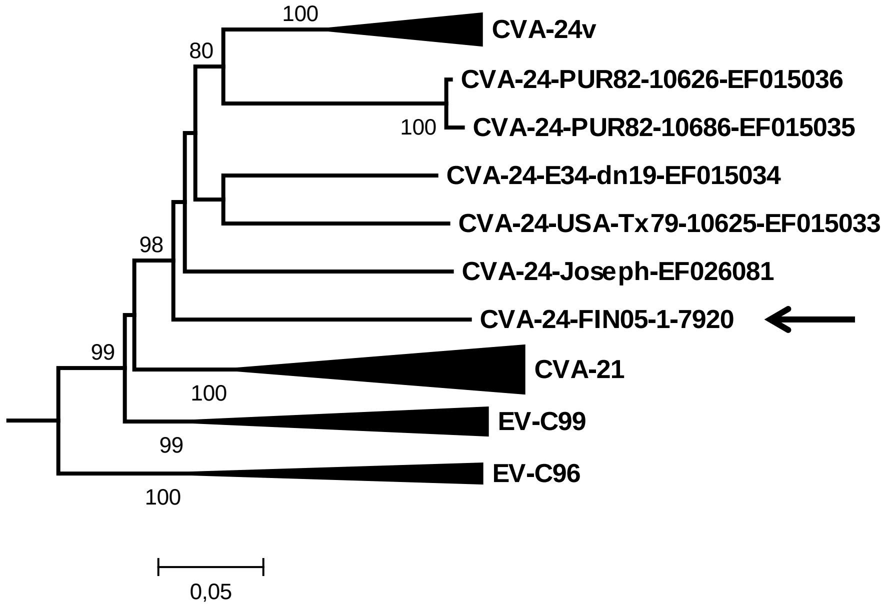

100
CVA-24v
80
CVA-24-PUR82-10626-EF015036
100
CVA-24-PUR82-10686-EF015035
CVA-24-E34-dn19-EF015034
CVA-24-USA-Tx79-10625-EF015033
98
CVA-24-Joseph-EF026081
CVA-24-FIN05-1-7920
99
CVA-21
100
EV-C99
99
EV-C96
100
0,05

(c) VP3

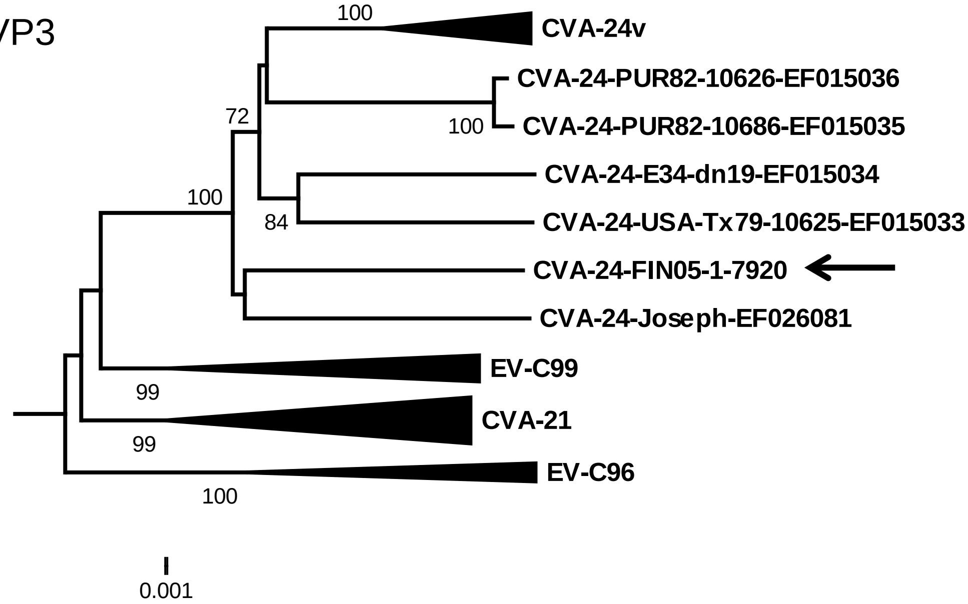

100
CVA-24v
CVA-24-PUR82-10626-EF015036
72
100
CVA-24-PUR82-10686-EF015035
100
CVA-24-E34-dn19-EF015034
84
CVA-24-USA-Tx79-10625-EF015033
CVA-24-FIN05-1-7920
CVA-24-Joseph-EF026081
EV-C99
99
CVA-21
99
EV-C96
100
0.001

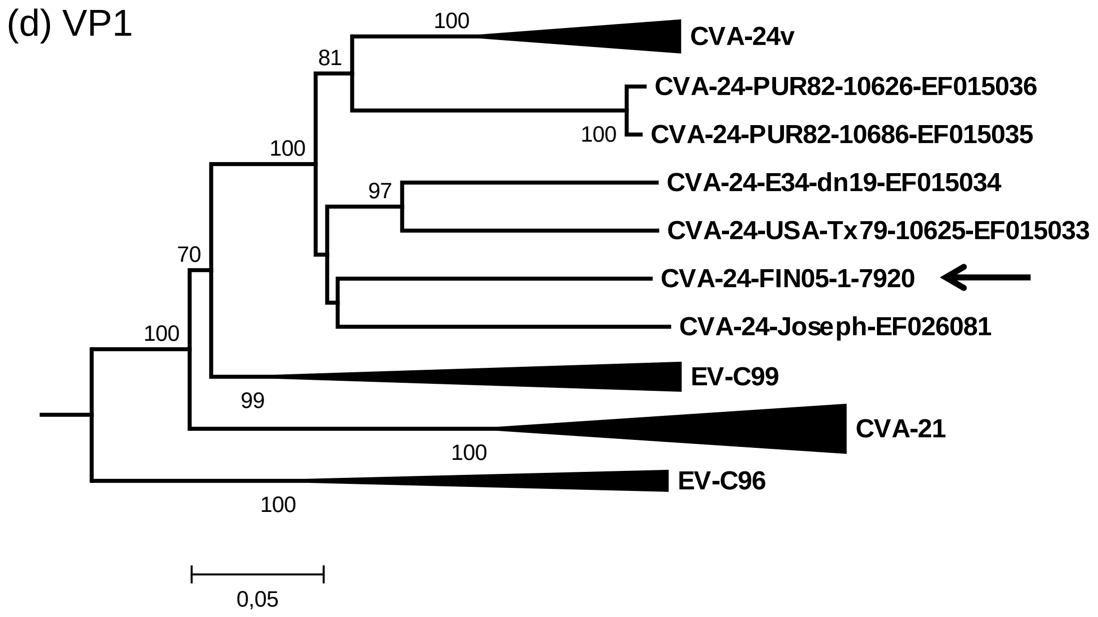
(d) VP1
100
CVA-24v
81
CVA-24-PUR82-10626-EF015036
100
CVA-24-PUR82-10686-EF015035
100
CVA-24-E34-dn19-EF015034
97
CVA-24-USA-Tx79-10625-EF015033
70
CVA-24-FIN05-1-7920
100
CVA-24-Joseph-EF026081
EV-C99
99
CVA-21
100
EV-C96
100
0,05

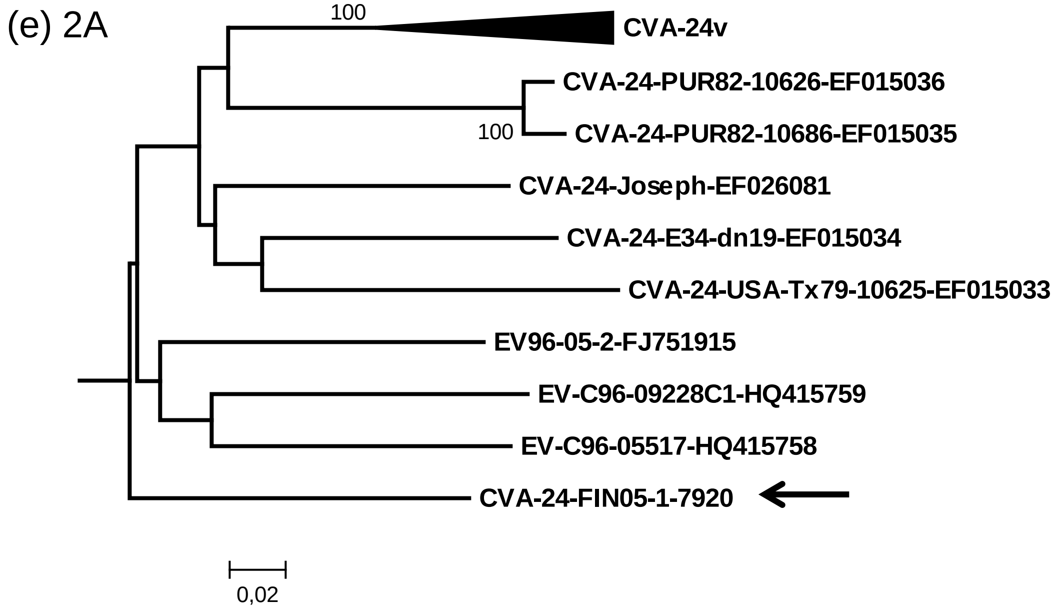
(e) 2A
100
CVA-24v
CVA-24-PUR82-10626-EF015036
100
CVA-24-PUR82-10686-EF015035
CVA-24-Joseph-EF026081
CVA-24-E34-dn19-EF015034
CVA-24-USA-Tx79-10625-EF015033
EV96-05-2-FJ751915
EV-C96-09228C1-HQ415759
EV-C96-05517-HQ415758
CVA-24-FIN05-1-7920
0,02

(f) 2B

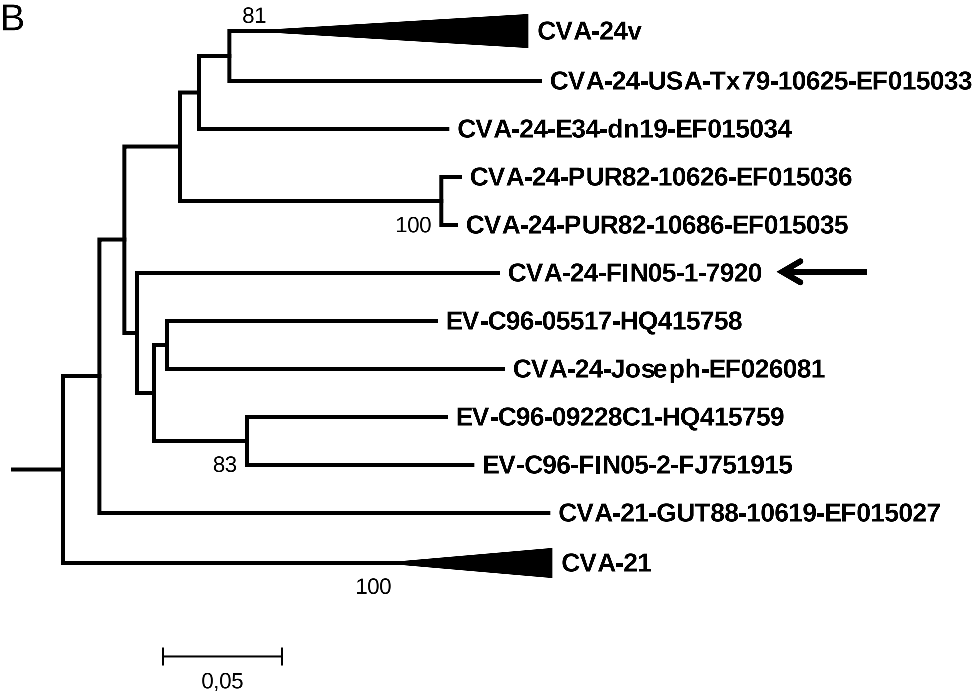
81
CVA-24v
CVA-24-USA-Tx79-10625-EF015033
CVA-24-E34-dn19-EF015034
CVA-24-PUR82-10626-EF015036
100
CVA-24-PUR82-10686-EF015035
CVA-24-FIN05-1-7920
EV-C96-05517-HQ415758
CVA-24-Joseph-EF026081
EV-C96-09228C1-HQ415759
83
EV-C96-FIN05-2-FJ751915
CVA-21-GUT88-10619-EF015027
CVA-21
100
0,05

(g) 2C

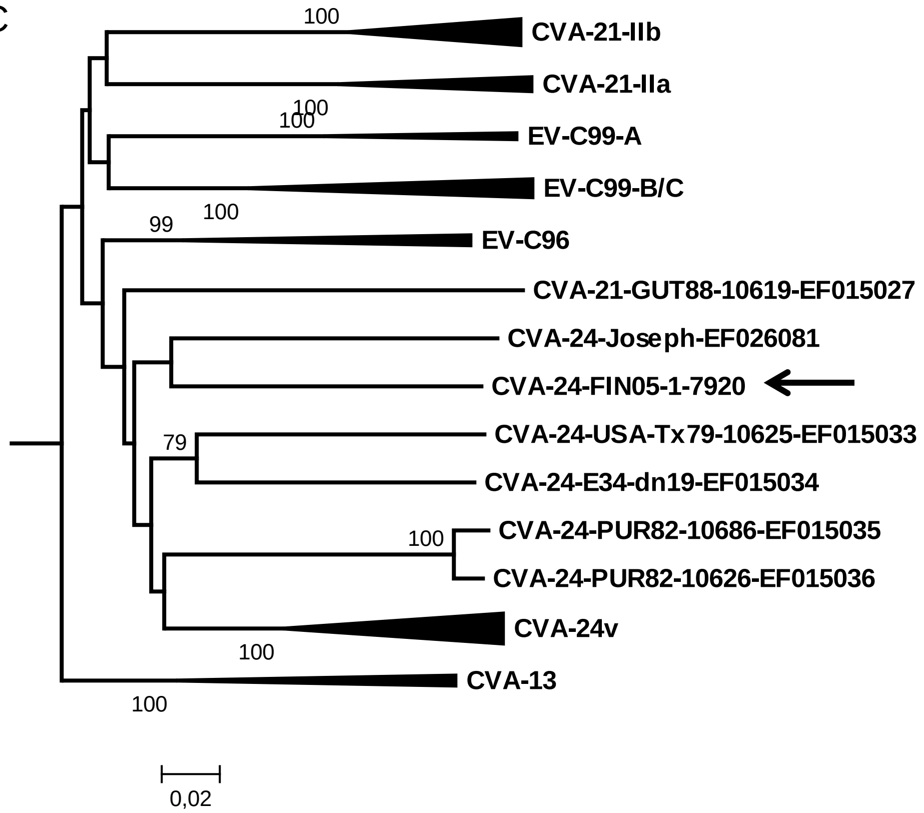
100
CVA-21-IIb
CVA-21-IIa
100
100
EV-C99-A
EV-C99-B/C
100
99
EV-C96
CVA-21-GUT88-10619-EF015027
CVA-24-Joseph-EF026081
CVA-24-FIN05-1-7920
79
CVA-24-USA-Tx79-10625-EF015033
CVA-24-E34-dn19-EF015034
100
CVA-24-PUR82-10686-EF015035
CVA-24-PUR82-10626-EF015036
CVA-24v
100
CVA-13
100
0,02

(h) 3A

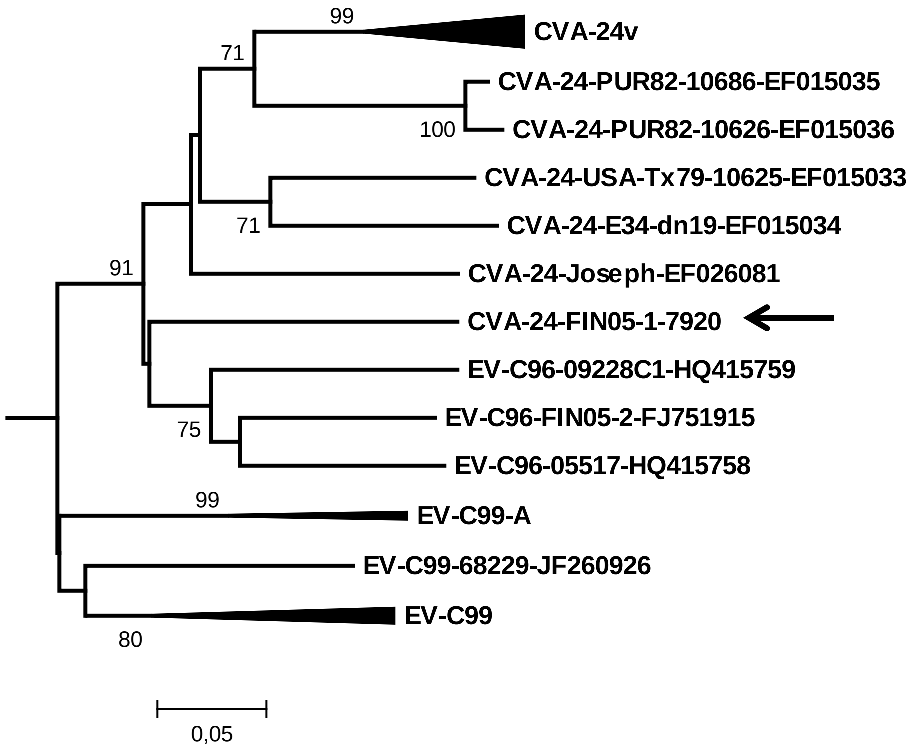
99
71
CVA-24v
CVA-24-PUR82-10686-EF015035
100
CVA-24-PUR82-10626-EF015036
CVA-24-USA-Tx79-10625-EF015033
71
CVA-24-E34-dn19-EF015034
91
CVA-24-Joseph-EF026081
CVA-24-FIN05-1-7920
EV-C96-09228C1-HQ415759
EV-C96-FIN05-2-FJ751915
75
EV-C96-05517-HQ415758
99
EV-C99-A
EV-C99-68229-JF260926
EV-C99
80
0,05

(i) 3C

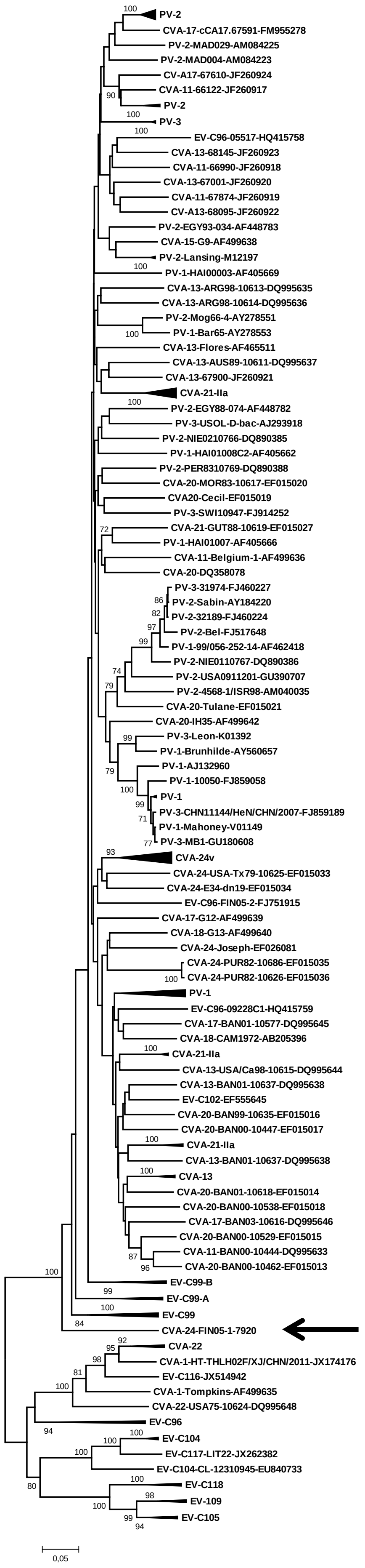
PV-2
CVA-17-cCA17.67591-FM955278
PV-2-MAD029-AM084225
PV-2-MAD004-AM084223
CV-A17-67610-JF260924
CVA-11-66122-JF260917
PV-2
PV-3
EV-C96-05517-HQ415758
CVA-13-68145-JF260923
CVA-11-66990-JF260918
CVA-13-67001-JF260920
CVA-11-67874-JF260919
CV-A13-68095-JF260922
PV-2-EGY93-034-AF448783
CVA-15-G9-AF499638
PV-2-Lansing-M12197
PV-1-HAI00003-AF405669
CVA-13-ARG98-10613-DQ995635
CVA-13-ARG98-10614-DQ995636
PV-2-Mog66-4-AY278551
PV-1-Bar65-AY278553
CVA-13-Flores-AF465511
CVA-13-AUS89-10611-DQ995637
CVA-13-67900-JF260921
CVA-21-IIa
PV-2-EGY88-074-AF448782
PV-3-USOL-D-bac-AJ293918
PV-2-NIE0210766-DQ890385
PV-1-HAI01008C2-AF405662
PV-2-PER8310769-DQ890388
CVA-20-MOR83-10617-EF015020
CVA20-Cecil-EF015019
PV-3-SWI10947-FJ914252
CVA-21-GUT88-10619-EF015027
PV-1-HAI01007-AF405666
CVA-11-Belgium-1-AF499636
CVA-20-DQ358078
PV-3-31974-FJ460227
PV-2-Sabin-AY184220
PV-2-32189-FJ460224
PV-2-Bel-FJ517648
PV-1-99/056-252-14-AF462418
PV-2-NIE0110767-DQ890386
PV-2-USA0911201-GU390707
PV-2-4568-1/ISR98-AM040035
CVA-20-Tulane-EF015021
CVA-20-IH35-AF499642
PV-3-Leon-K01392
PV-1-Brunhilde-AY560657
PV-1-AJ132960
PV-1-10050-FJ859058
PV-1
PV-3-CHN11144/HeN/CHN/2007-FJ859189
PV-1-Mahoney-V01149
PV-3-MB1-GU180608
CVA-24v
CVA-24-USA-Tx79-10625-EF015033
CVA-24-E34-dn19-EF015034
EV-C96-FIN05-2-FJ751915
CVA-17-G12-AF499639
CVA-18-G13-AF499640
CVA-24-Joseph-EF026081
CVA-24-PUR82-10686-EF015035
CVA-24-PUR82-10626-EF015036
PV-1
EV-C96-09228C1-HQ415759
CVA-17-BAN01-10577-DQ995645
CVA-18-CAM1972-AB205396
CVA-21-IIa
CVA-13-USA/Ca98-10615-DQ995644
CVA-13-BAN01-10637-DQ995638
EV-C102-EF555645
CVA-20-BAN99-10635-EF015016
CVA-20-BAN00-10447-EF015017
CVA-21-IIa
CVA-13-BAN01-10637-DQ995638
CVA-13
CVA-20-BAN01-10618-EF015014
CVA-20-BAN00-10538-EF015018
CVA-17-BAN03-10616-DQ995646
CVA-20-BAN00-10529-EF015015
CVA-11-BAN00-10444-DQ995633
CVA-20-BAN00-10462-EF015013
EV-C99-B
EV-C99-A
EV-C99
CVA-24-FIN05-1-7920
CVA-22
CVA-1-HT-THLH02F/XJ/CHN/2011-JX174176
EV-C116-JX514942
CVA-1-Tompkins-AF499635
CVA-22-USA75-10624-DQ995648
EV-C96
EV-C104
EV-C117-LIT22-JX262382
EV-C104-CL-12310945-EU840733
EV-C118
EV-109
EV-C105
0,05

(j) 3D

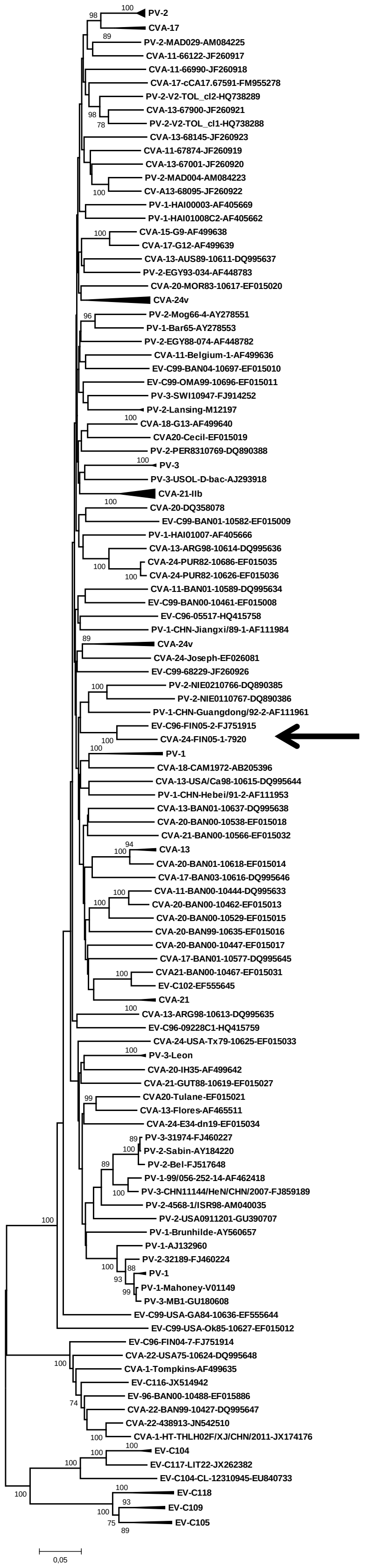

Supplementary Figure S1. The phylogenetic trees constructed from distinct genes of EV-C strains, of which a complete genome sequence was available. The trees were constructed using the Neighbour-Joining method and the Tamura-Nei substitution model. The bootstrap support values were calculated for 1000 replicates. The bootstrap support values > 70 are shown.
